# Bis(μ-1,4-di­hydro­pyridin-4-one-κ^2^*O*:*O*)di-μ-perchlorato-κ^4^*O*:*O*′-bis­[aqua­(1,4-di­hydro­pyridin-4-one-κ*O*)copper(II)] tetra­aqua­bis­(1,4-di­hydropyridin-4-one-κ*O*)copper(II) tetra­kis­(perchlorate) 1,4-di­hydro­pyridin-4-one disolvate

**DOI:** 10.1107/S2414314626003494

**Published:** 2026-04-14

**Authors:** Mark M. Turnbull, Christopher P. Landee, Jan L. Wikaira

**Affiliations:** a Carlson School of Chemistry and Biochemistry, Clark University, 950 Main St., Worcester, MA 01610, USA; b Dept. of Physics, Clark University, 950 Main St., Worcester, MA 01610, USA; c School of Physical and Chemical Sciences, University of Canterbury, Private Bag 4800, Christchurch, New Zealand; Purdue University, USA

**Keywords:** crystal structure, Cu(II), 4-pyridone, co-crystal

## Abstract

The reaction of copper(II) perchlorate hexa­hydrate with 4-pyridone and pyrazine in 1-propanol serendipitously yielded crystals of the solvated title double salt, [Cu(C_5_H_5_NO)_2_(H_2_O)_4_][Cu_2_(ClO_4_)_2_(C_5_H_5_NO)_4_(H_2_O)_2_](ClO_4_)_4_·2C_5_H_5_NO, as a biproduct. The copper cations in both the monometallic complex and the dimeric species exhibit classic Jahn-Teller-like elongations. All coordinating 4-pyridone mol­ecules are in the κ-*O* coordination mode. The dimeric component exhibits both bridging 4-pyridone and perchlorate ligands. Both complexes and the 4-pyridone mol­ecules are bound together by a network of classical hydrogen bonds. The non-coordinating perchlorate ions were modeled as three-site disordered and one of the coordinating 4-pyridone mol­ecules was modeled as two-site disordered.

## Abstract 2

The reaction of copper(II) perchlorate hexa­hydrate with 4-pyridone and pyrazine in 1-propanol serendipitously yielded crystals of the solvated title double salt as a byproduct. Both the monometallic Cu complex and the dimeric species exhibit classic Jahn-Teller-like elongations. Both complexes and the 4-pyridone mol­ecules are bound together by a network of classical hydrogen bonds.

## Graphical abstract

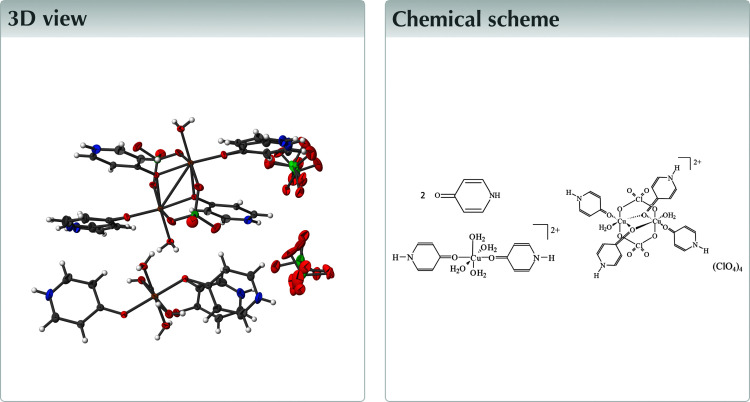

## Structure description

2- and 4-hy­droxy­pyridines and their corresponding pyridones exist in tautomeric equilibrium and aspects of their coordination chemistry have been reviewed (Rawson & Winpenny, 1995 ▸). Although normally it is the κ-*O*-pyridone form that coordinates to first-row transition-metal ions, exceptions are known where it is the κ-*N*-hy­droxy­pyridine form that is observed, or even both (Graci *et al.*, 2024 ▸). In one exceptional case involving 2-bromo-4-hy­droxy­pyridine, a complex was isolated where both tautomers exist coordinating to a Cu^II^ ion, and the crystal contains both tautomers as well (Monroe & Turnbull, 2019 ▸). In the course of our studies of pyrazine-bridged Cu^II^ chains with pyridones as ancilliary ligands (Monroe *et al.*, 2024 ▸; Kirkman-Davis *et al.*, 2020 ▸), we serendipitously isolated the title crystal, which incorporates two different pyridone-coordinated copper cations as well as solvent pyridone mol­ecules.

**Figure 1:**
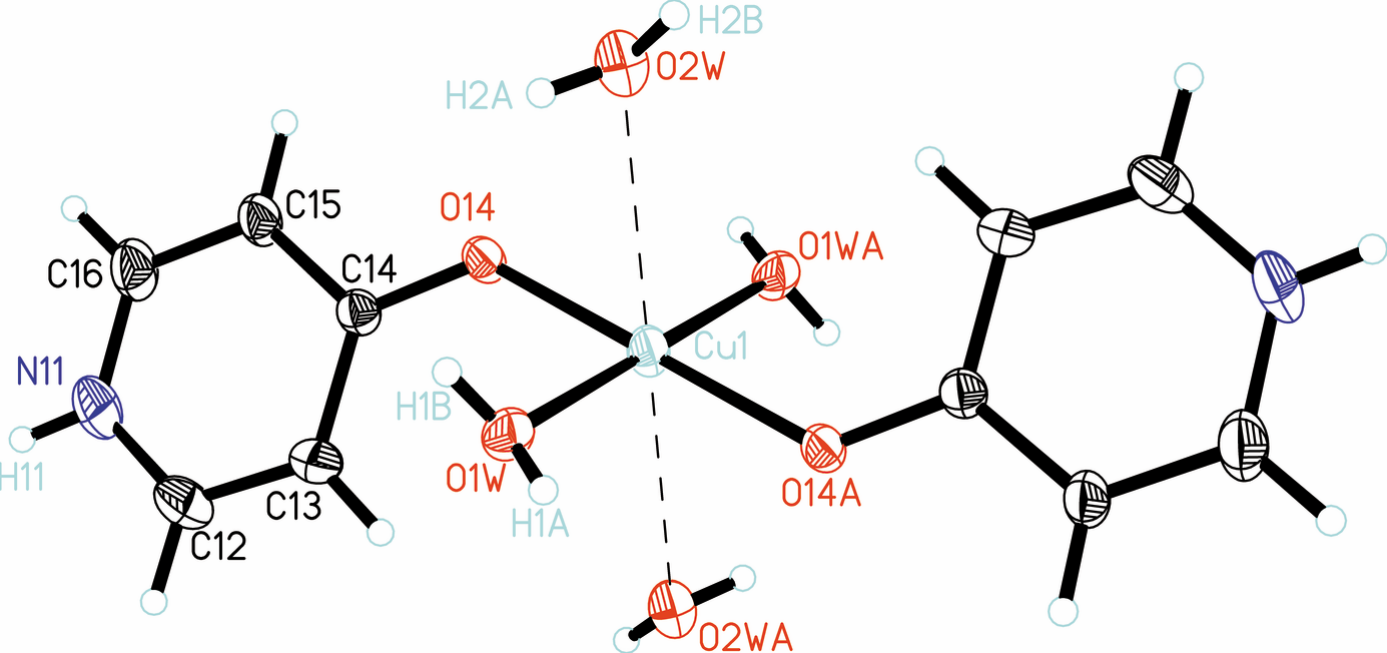
The mol­ecule [Cu((H_2_O)_4_(4-pyridone)_2_](ClO_4_)_2_ shown as 50% probability ellipsoids with hydrogen atoms shown as spheres of arbitrary size. Only the asymmetric unit, copper coordination sphere and those hydrogen atoms whose positions were refined are labeled.

The title crystal comprises a double salt of [Cu(H_2_O)(4-pyridone)(μ-4-pyridone)(μ-ClO_4_)]_2_(ClO_4_)_2_ and [Cu((H_2_O)_4_(4-pyridone)_2_](ClO_4_)_2_ and two 4-pyridone solvent mol­ecules. Selected bond lengths and angles are presented in Table 1 ▸. All coordinating 4-pyridone mol­ecules exhibit κ-*O* (or μ-κ-*O*)coordination modes, and the solvent mol­ecules are in the 4-pyridone tautomer, rather than the hy­droxy­pyridine tautomer. The Cu^II^ ion of the [Cu((H_2_O)_4_(4-pyridone)_2_](ClO_4_)_2_ mol­ecule sits on a crystallographic inversion center with one 4-pyridone mol­ecule and two water mol­ecules constituting the asymmetric unit (Fig. 1 ▸). It may be thought of as a very highly Jahn–Teller-like elongated octa­hedron with two 4-pyridone mol­ecules and two water mol­ecules lying in the equatorial plane. The remaining Cu—O bond (Cu1—O2*W*) is exceedingly long [2.8760 (12) Å], but lies only 12.5° from the normal to that plane, indicating that its location is not accidental. A quick search of the Cambridge Structural Database (CSD; Groom *et al.*, 2016 ▸) indicates nearly 100 structures with CuO_6_ polyhedra with a pair of Cu—O bonds between 2.7–3.0 Å in length. Charge balance is achieved *via* two perchlorate anions.

**Figure 2:**
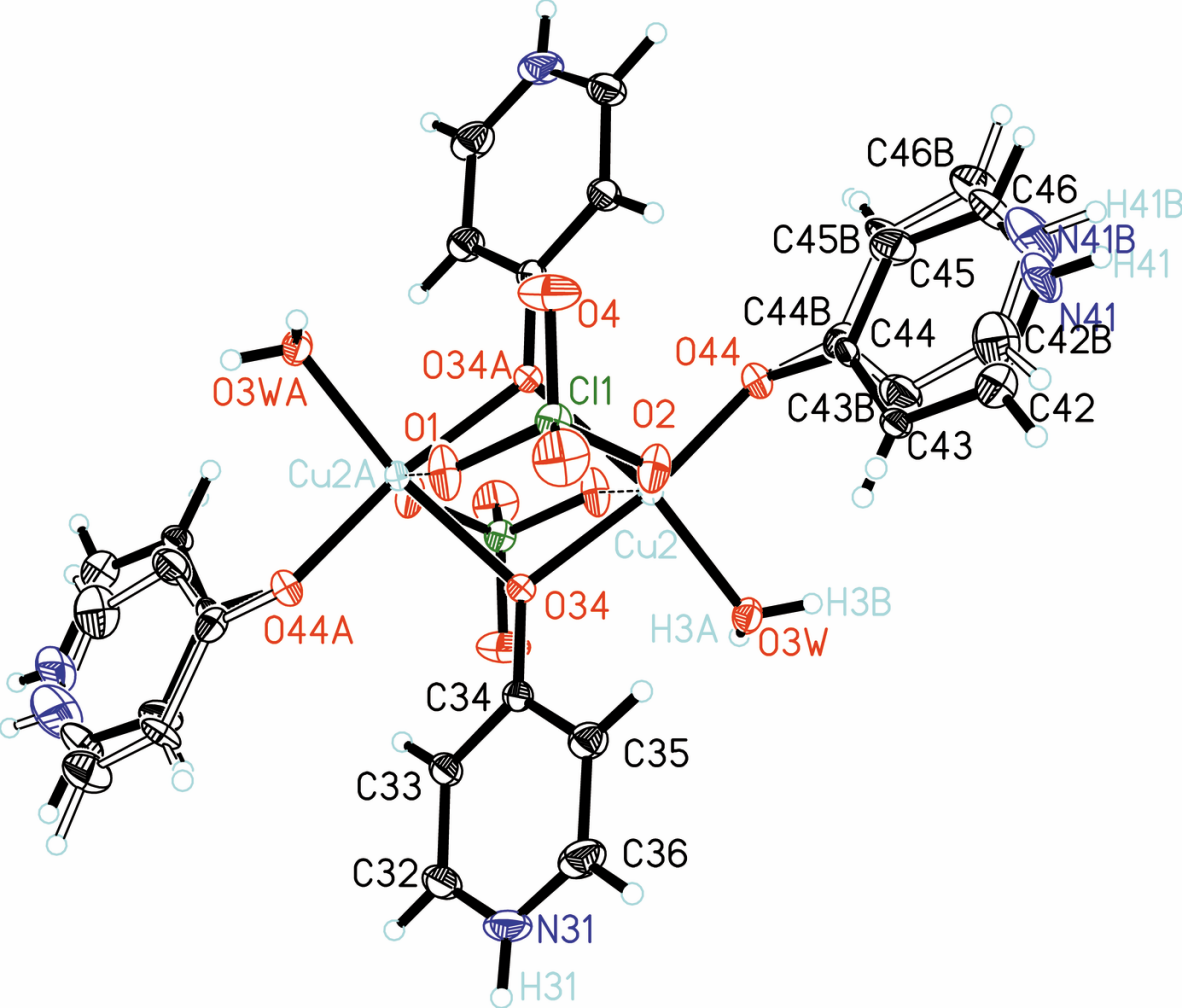
The mol­ecule [Cu(H_2_O)(4-pyridone)(μ-4-pyridone)(μ-ClO_4_)]_2_(ClO_4_)_2_ drawn with displacement ellipsoids at the 50% probability level with hydrogen atoms shown as spheres of arbitrary size. Only the asymmetric unit, copper coordination sphere and those hydrogen atoms whose positions were refined are labeled.

The [Cu(H_2_O)(4-pyridone)(μ-4-pyridone)(μ-ClO_4_)]_2_(ClO_4_)_2_ mol­ecule lies athwart a second inversion center located midway between the two Cu^II^ ions (Fig. 2 ▸). The asymmetric unit comprises one terminal 4-pyridone mol­ecule, one water mol­ecule, one bridging 4-pyridone mol­ecule, one bridging perchlorate ion and one non-coordinating perchlor­ate anion per copper(II). The Cu—O—Cu bridge is nearly symmetrical [*d*_Cu—O_ = 1.9534 (10), 1.9943 (9) Å]. The coordination environment is again well described by a classic Jahn–Teller-like elongation where the water mol­ecule, and the bridging and terminal 4-pyridone mol­ecules form the equatorial plane (mean deviation of those atoms from the plane including Cu2 = 0.0395 Å). The axial positions are occupied by bridging perchlorate ions with Cu—O distances of 2.4885 (10) Å [O1(1 − *x*, −*y*, 1 − *z*)] and 2.5898 (10) Å (O2). Such bis-perchlorate bridges in copper(II) complexes are well known, frequently with bis-hydroxide bridges accompanying them, such as observed in bis­(μ-hydroxido)bis­(μ-perchlorato)tetra­kis­(2-amino-4-methyl­pyrimidine)­dicopper(II) (Am­ani Komaei *et al.*, 1999 ▸) and bis­[(μ-hydroxido)(μ-perchlorato-*O*,*O*′)(di-2-pyridyl­amine)­cop­per(II)] (Youngme *et al.*, 2002 ▸), although those with alk­oxy bridges such as bis­(μ-perchlorato)bis­(μ-methoxo)tetra­kis­(2-methyl­pyrazine)­dicopper(II) (Ar­aujo-Martinez *et al.*, 2023 ▸) and phenoxide bridges such as bis­{μ-[2-({[2-(3,5-dimethyl-1*H*-pyrazol-1-yl)eth­yl]imino}­meth­yl) phenolato]}bis­(μ-per­chlorato)dicopper(II) (Maria *et al.*, 2020 ▸) are also known. The terminal 4-pyridone mol­ecule is two-site disordered, roughly about the carbonyl axis. Refined occupancies for the two rings are nearly 47:53 with the N41—H41/N41*A*—H41*A* moieties serving as hydrogen-bond donors to one of the disordered non-coordinating perchlorate anions.

**Figure 3:**
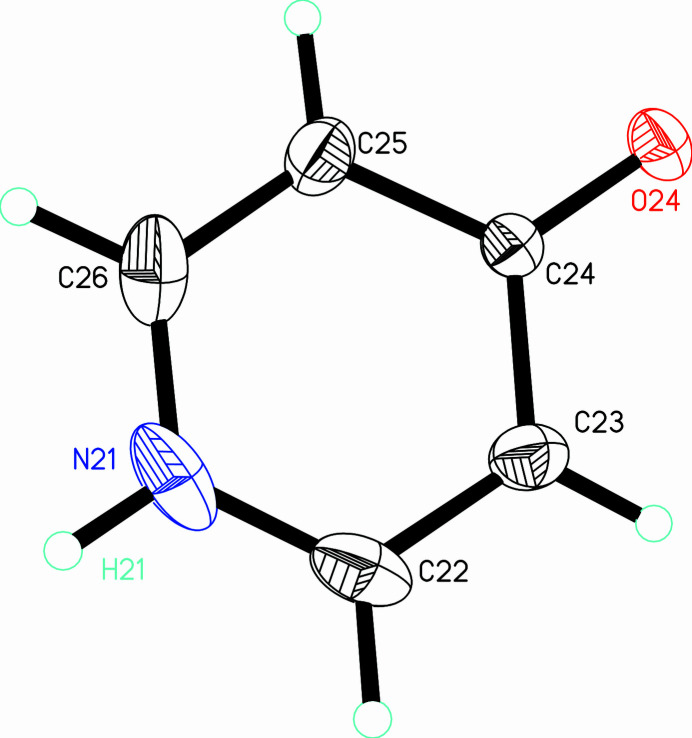
The solvent 4-pyridone molecule drawn with displacement ellipsoids at the 50% probability level with hydrogen atoms shown as spheres of arbitrary size. Only the hydrogen atom whose position was refined is labeled.

Finally, there is a solvent 4-pyridone mol­ecule containing N21 (Fig. 3 ▸). It appears in the pyridone tautomer and is stabilized in the crystal *via* hydrogen bonds (*see below*).

The length of the C=O bonds in the pyridone mol­ecules varies from 1.2885 (16) Å (C24=O24) to 1.3254 (14) Å (C34=O34), indicating a variation in the double-bond strength. The shortest is in the non-coordinating pyridone mol­ecule where the carbonyl is subject only to inter­molecular hydrogen bonding (*see below*) and the longest is in the μ-pyridone mol­ecule where there are two Cu—O bonds to the carbonyl oxygen. The C24=O24 bond is slightly longer than observed in either reported polymorph of the free ligand [*d* = 1.274 Å (*C*2/*c*) = 1.269 Å (*Pbca*)] (Tyl *et al.*, 2008 ▸), which may result from it serving as a hydrogen-bond acceptor for two donors in the current compound rather than one in the free ligand. However, the C24=O24 bond is also longer than observed in 4-pyridone hydrate, a complex structure with five independent pyridone mol­ecules, where the average C=O bond is 1.272 (1) Å and all carbonyls serve as hydrogen-bond acceptors to two donors, whether water mol­ecules or other pyridones (Jones, 2001 ▸). The C34=O34 bond of the bridging pyridone mol­ecule is longer than observed in *catena*-[(μ-pyrazine)­bis­(μ-4-pyridone)bis­(μ-hydroxido)di­cop­per(II)] bis­(perchlorate) (1.306 Å; Mukda *et al.*, 2024 ▸); however, in that complex the pyridone bridge is far from symmetric (Cu—O = 1.845/2.300 Å).

**Figure 4:**
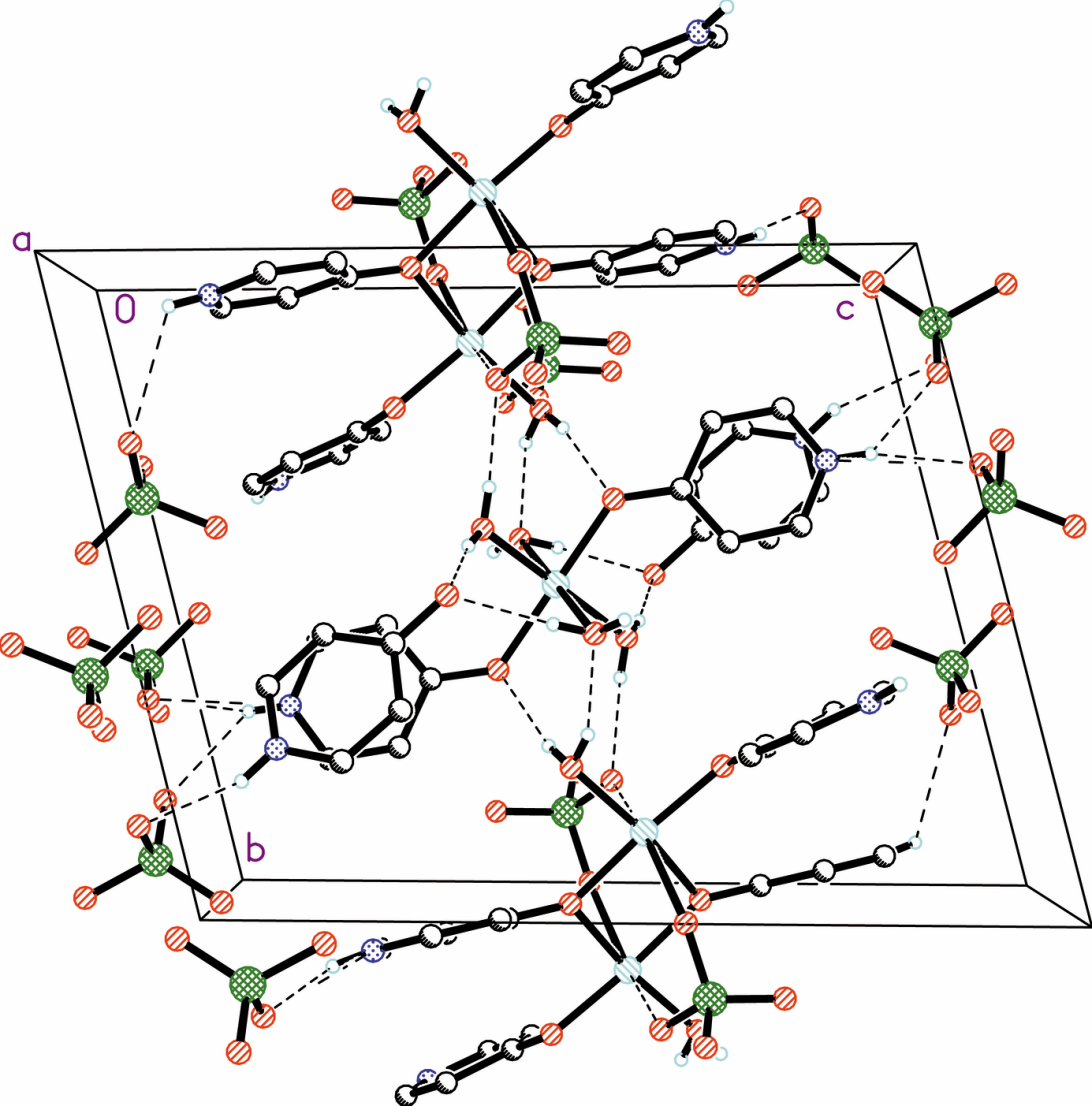
Packing diagram of the crystal viewed parallel to the *a*-axis. Dashed lines represent hydrogen bonds. Only the major component of disordered mol­ecules/ions and only those hydrogen atoms involved in classical hydrogen bonds are shown.

The crystal structure is supported by a myriad of hydrogen bonds (Table 2 ▸ and Fig. 4 ▸). The pyridone N—H functionalities serve as donors with the perchlorate ions serving as acceptors. O1*W* (coordinating to Cu1) serves as an hydrogen-bond donor to a perchlorate ion and to the carbonyl oxygen of the solvent 4-pyridone mol­ecule. O2*W* also provides hydrogen bonds to two solvent 4-pyridone mol­ecule and serves as an acceptor for a hydrogen bond from O3*W*. Finally, O3*W* also serves as a hydrogen-bond donor to carbonyl oxygen O14, the only coordinating pyridone oxygen (bonded to Cu1) serves as an acceptor.

## Synthesis and crystallization

The title crystal was isolated as a by-product of the synthesis of [Cu(pz)(4-pyridone)_2_(H_2_O)_2_](ClO_4_)_2_. Copper(II) perchlorate hexa­hydrate, pyrazine (pz) and 4-hy­droxy­pyridine were dissolved in 1-propanol in a 1:1:2 molar ratio and left for slow evaporation. After three weeks, blue crystals of [Cu(pz)(4-pyridone)_2_(H_2_O)_2_](ClO_4_)_2_ were isolated by filtration. A few small off-aqua colored crystals were separated by hand. Single-crystal X-ray analysis showed them to be the title material.

## Refinement

Crystal data, data collection and structure refinement details are summarized in Table 3 ▸. Hydrogen atoms bonded to carbon atoms were placed geometrically and refined with fixed isotropic displacement parameters with *d*(C—H) = 0.93 Å, *U*_iso_(H) = 1.2*U*_eq_(C). Hydrogen atoms bonded to N or O atoms were located in the difference map and their positions refined using anti­bumping restraints. O—H and N—H distances were restrained to be at least 0.082 (2) and 0.085 (2) Å, respectively, and refined with fixed isotropic displacement parameters [*U*_iso_(H) = 1.2*U*_eq_(N or O)]. One of the 4-pyridone rings (containing N41) was modeled as two-site disordered roughly about the O44—C44 axis and was refined using SIMU and SAME restraints for the two portions. The O atoms O44/O44*B* were omitted from the disorder. Final refined occupancies were 0.466 (10):0.534 (10) for the N41 and N41*A* rings, respectively. Two of the perchlorate ions (containing Cl2/Cl2*A*/Cl2*B* and Cl3/Cl3*A*/Cl3*B*) were modeled as three-site disordered. The ions were refined with SIMU and SAME restraints. Refined occupancies for the ions were: Cl2/Cl2*A*/Cl2*B*, 0.428 (3): 0.429 (3): 0.136 (3) and Cl3/Cl3*A*/Cl3*B*, 0.479 (9): 0.489 (9): 0.038 (2) respectively.

## Supplementary Material

Crystal structure: contains datablock(s) I. DOI: 10.1107/S2414314626003494/zl4096sup1.cif

Structure factors: contains datablock(s) I. DOI: 10.1107/S2414314626003494/zl4096Isup3.hkl

CCDC reference: 2543656

Additional supporting information:  crystallographic information; 3D view; checkCIF report

## Figures and Tables

**Table 1:** Selected geometric parameters (Å, °)

Cu1—O14	1.9276 (9)	Cu2—O34^i^	1.9943 (9)
Cu1—O1*W*	1.9501 (11)	Cu2—O1^i^	2.4885 (10)
Cu1—O2*W*	2.8760 (12)	Cu2—O2	2.5898 (10)
C14—O14	1.2965 (15)	C34—O34	1.3254 (14)
Cu2—O44*B*	1.8895 (9)	C44—O44	1.313 (7)
Cu2—O44	1.8895 (9)	C44*B*—O44*B*	1.273 (7)
Cu2—O3*W*	1.9534 (10)	C24—O24	1.2885 (16)
Cu2—O34	1.9534 (9)		
			
O14—Cu1—O1*W*	91.08 (4)	O44—Cu2—O1^i^	90.09 (4)
O14—Cu1—O2*W*	80.65 (4)	O3*W*—Cu2—O1^i^	88.40 (4)
O1*W*—Cu1—O2*W*	81.98 (4)	O34—Cu2—O1^i^	84.16 (4)
O44*B*—Cu2—O3*W*	95.60 (4)	O34^i^—Cu2—O1^i^	82.98 (4)
O44—Cu2—O3*W*	95.60 (4)	O44*B*—Cu2—O2	97.32 (4)
O44—Cu2—O34	170.44 (4)	O44—Cu2—O2	97.32 (4)
O3*W*—Cu2—O34	91.88 (4)	O3*W*—Cu2—O2	105.16 (4)
O44*B*—Cu2—O34^i^	92.58 (4)	O34—Cu2—O2	86.43 (3)
O3*W*—Cu2—O34^i^	168.13 (4)	O34^i^—Cu2—O2	82.24 (3)
O34—Cu2—O34^i^	79.16 (4)	O1^i^—Cu2—O2	163.73 (3)

Symmetry code: (i) 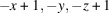.

**Table 2:** Hydrogen-bond geometry (Å, °)

*D*—H⋯*A*	*D*—H	H⋯*A*	*D*⋯*A*	*D*—H⋯*A*
N11—H11⋯O81^ii^	0.82 (2)	2.06 (2)	2.858 (9)	164 (2)
N11—H11⋯O81*A*^ii^	0.82 (2)	2.26 (2)	2.961 (3)	143 (2)
O1*W*—H1*A*⋯O1^iii^	0.78 (2)	1.99 (2)	2.7695 (15)	176 (2)
O1*W*—H1*B*⋯O24	0.79 (1)	1.83 (2)	2.6124 (15)	171 (2)
O2*W*—H2*A*⋯O24	0.79 (1)	1.99 (1)	2.7753 (14)	172 (2)
O2*W*—H2*B*⋯O24^iv^	0.83 (2)	2.07 (2)	2.8705 (16)	161 (2)
O3*W*—H3*A*⋯O14	0.79 (1)	1.94 (1)	2.6941 (13)	161 (2)
O3*W*—H3*B*⋯O2*W*	0.79 (1)	1.95 (2)	2.6875 (15)	157 (2)
N31—H31⋯O6^v^	0.84 (1)	2.09 (2)	2.882 (9)	156 (2)
N31—H31⋯O5*A*^v^	0.84 (1)	2.12 (2)	2.928 (9)	160 (2)
N41—H41⋯O83^vi^	0.84 (2)	2.16 (2)	2.974 (6)	163 (5)
N41*B*—H41*B*⋯O82*A*^vi^	0.89 (5)	2.25 (5)	3.104 (6)	161 (4)
N41*B*—H41*B*⋯O83*A*^vi^	0.89 (5)	2.39 (5)	3.109 (14)	138 (4)
N21—H21⋯O6	0.84 (2)	2.31 (2)	3.013 (9)	142 (2)
N21—H21⋯O5*A*	0.84 (2)	2.28 (2)	3.013 (9)	147 (2)
N21—H21⋯O7*A*^v^	0.84 (2)	2.59 (2)	3.263 (7)	138 (2)

Symmetry codes: (ii) 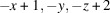; (iii) 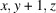; (iv) 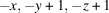; (v) 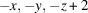; (vi) 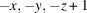.

**Table 3:** Experimental details

Crystal data	
Chemical formula	[Cu(C_5_H_5_NO)_2_(H_2_O)_4_]·[Cu_2_(ClO_4_)_2_(C_5_H_5_NO)_4_(H_2_O)_2_](ClO_4_)_4_·2C_5_H_5_NO
*M*_r_	1656.21
Crystal system, space group	Triclinic, *P* 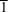
Temperature (K)	123
*a*, *b*, *c* (Å)	8.2317 (4), 12.2025 (5), 15.5562 (5)
α, β, γ (°)	75.730 (3), 85.216 (3), 87.906 (3)
*V* (Å^3^)	1508.88 (10)
*Z*	1
Radiation type	Mo *K*α
μ (mm^−1^)	1.42
Crystal size (mm)	0.40 × 0.30 × 0.30

Data collection	
Diffractometer	Xcalibur, Ruby, Gemini
Absorption correction	Multi-scan (*CrysAlis PRO*; Agilent, 2013 ▸)
*T*_min_, *T*_max_	0.839, 1.000
No. of measured, independent and observed [*I* > 2σ(*I*)] reflections	30101, 14629, 11280
*R*_int_	0.030
(sin θ/λ)_max_ (Å^−1^)	0.833

Refinement	
*R*[*F*^2^ > 2σ(*F*^2^)], *wR*(*F*^2^), *S*	0.037, 0.089, 1.02
No. of reflections	14629
No. of parameters	705
No. of restraints	1128
H-atom treatment	H atoms treated by a mixture of independent and constrained refinement
Δρ_max_, Δρ_min_ (e Å^−3^)	0.51, −0.50

Computer programs: *CrysAlis PRO* (Agilent, 2013 ▸), *SHELXS2014* (Sheldrick, 2008 ▸) and *SHELXL2019/2* (Sheldrick, 2015 ▸).

## full crystallographic data

### Bis(µ-1,4-dihydropyridin-4-one-κ2O:O)di-µ-perchlorato-κ4O:O'-bis[aqua(1,4-dihydropyridin-4-one-κO)copper(II)] tetraaquabis(1,4-dihydropyridin-4-one-κO)copper(II) tetrakis(perchlorate) 1,4-dihydropyridin-4-one disolvate Crystal data

**Table:**

[Cu(C_5_H_5_NO)_2_(H_2_O)_4_]·[Cu_2_(ClO_4_)_2_(C_5_H_5_NO)_4_(H_2_O)_2_](ClO_4_)_4_·2C_5_H_5_NO	*Z* = 1
*M**_r_* = 1656.21	*F*(000) = 841
Triclinic, *P*1	*D*_x_ = 1.823 Mg m^−^^3^
*a* = 8.2317 (4) Å	Mo *K*α radiation, λ = 0.71073 Å
*b* = 12.2025 (5) Å	Cell parameters from 10098 reflections
*c* = 15.5562 (5) Å	θ = 3.2–40.1°
α = 75.730 (3)°	µ = 1.42 mm^−^^1^
β = 85.216 (3)°	*T* = 123 K
γ = 87.906 (3)°	Block, blue-green
*V* = 1508.88 (10) Å^3^	0.40 × 0.30 × 0.30 mm

### Bis(µ-1,4-dihydropyridin-4-one-κ2O:O)di-µ-perchlorato-κ4O:O'-bis[aqua(1,4-dihydropyridin-4-one-κO)copper(II)] tetraaquabis(1,4-dihydropyridin-4-one-κO)copper(II) tetrakis(perchlorate) 1,4-dihydropyridin-4-one disolvate Data collection

**Table:**

Xcalibur, Ruby, Gemini diffractometer	11280 reflections with *I* > 2σ(*I*)
ω scans	*R*_int_ = 0.030
Absorption correction: multi-scan (CrysAlisPro; Agilent, 2013)	θ_max_ = 36.3°, θ_min_ = 3.0°
*T*_min_ = 0.839, *T*_max_ = 1.000	*h* = −13→13
30101 measured reflections	*k* = −20→20
14629 independent reflections	*l* = −25→13

### Bis(µ-1,4-dihydropyridin-4-one-κ2O:O)di-µ-perchlorato-κ4O:O'-bis[aqua(1,4-dihydropyridin-4-one-κO)copper(II)] tetraaquabis(1,4-dihydropyridin-4-one-κO)copper(II) tetrakis(perchlorate) 1,4-dihydropyridin-4-one disolvate Refinement

**Table:**

Refinement on *F*^2^	Secondary atom site location: difference Fourier map
Least-squares matrix: full	Hydrogen site location: mixed
*R*[*F*^2^ > 2σ(*F*^2^)] = 0.037	H atoms treated by a mixture of independent and constrained refinement
*wR*(*F*^2^) = 0.089	*w* = 1/[σ^2^(*F*_o_^2^) + (0.0353*P*)^2^ + 0.0761*P*] where *P* = (*F*_o_^2^ + 2*F*_c_^2^)/3
*S* = 1.02	(Δ/σ)_max_ = 0.001
14629 reflections	Δρ_max_ = 0.51 e Å^−^^3^
705 parameters	Δρ_min_ = −0.50 e Å^−^^3^
1128 restraints	Extinction correction: SHELXL-2019/2 (Sheldrick 2015), Fc^*^=kFc[1+0.001xFc^2^λ^3^/sin(2θ)]^-1/4^
Primary atom site location: structure-invariant direct methods	Extinction coefficient: 0.0050 (4)

### Bis(µ-1,4-dihydropyridin-4-one-κ2O:O)di-µ-perchlorato-κ4O:O'-bis[aqua(1,4-dihydropyridin-4-one-κO)copper(II)] tetraaquabis(1,4-dihydropyridin-4-one-κO)copper(II) tetrakis(perchlorate) 1,4-dihydropyridin-4-one disolvate Special details

**Table:**

Geometry. All esds (except the esd in the dihedral angle between two l.s. planes) are estimated using the full covariance matrix. The cell esds are taken into account individually in the estimation of esds in distances, angles and torsion angles; correlations between esds in cell parameters are only used when they are defined by crystal symmetry. An approximate (isotropic) treatment of cell esds is used for estimating esds involving l.s. planes.
Refinement. Data collection was carried out with an Oxford Diffraction Xcalibur diffractometer employing Mo Kα radiation (λ = 1.5418 Å). *CrysAlis PRO* (Agilent, 2013) was utilized for data collection, cell refinement and data reduction. The structure was solved using *SHELXS2014* (Sheldrick, 2008) and refined using *SHELXL2019*/3 (Sheldrick, 2015).

### Bis(µ-1,4-dihydropyridin-4-one-κ2O:O)di-µ-perchlorato-κ4O:O'-bis[aqua(1,4-dihydropyridin-4-one-κO)copper(II)] tetraaquabis(1,4-dihydropyridin-4-one-κO)copper(II) tetrakis(perchlorate) 1,4-dihydropyridin-4-one disolvate Fractional atomic coordinates and isotropic or equivalent isotropic displacement parameters (Å^2^)

**Table:**

	*x*	*y*	*z*	*U*_iso_*/*U*_eq_	Occ. (<1)
Cu1	0.500000	0.500000	0.500000	0.02113 (6)	
N11	0.53187 (19)	0.30642 (14)	0.86036 (8)	0.0333 (3)	
H11	0.540 (3)	0.2965 (18)	0.9140 (10)	0.040*	
C12	0.5681 (2)	0.40905 (16)	0.80839 (10)	0.0329 (4)	
H12	0.603951	0.465660	0.834497	0.040*	
C13	0.55457 (19)	0.43384 (13)	0.71886 (9)	0.0243 (3)	
H13	0.582329	0.506618	0.682898	0.029*	
C14	0.49865 (17)	0.34995 (11)	0.67988 (8)	0.0182 (2)	
O14	0.48104 (13)	0.36529 (8)	0.59567 (6)	0.02038 (19)	
C15	0.4625 (2)	0.24333 (12)	0.73767 (8)	0.0238 (3)	
H15	0.425193	0.184724	0.714193	0.029*	
C16	0.4802 (2)	0.22329 (14)	0.82629 (9)	0.0299 (3)	
H16	0.456331	0.150954	0.864214	0.036*	
O1W	0.36469 (14)	0.58573 (9)	0.57020 (7)	0.0218 (2)	
H1A	0.352 (2)	0.6502 (13)	0.5501 (11)	0.026*	
H1B	0.279 (2)	0.5594 (16)	0.5911 (11)	0.026*	
O2W	0.18710 (14)	0.42539 (9)	0.46968 (7)	0.0235 (2)	
H2A	0.151 (2)	0.4394 (16)	0.5143 (11)	0.028*	
H2B	0.119 (2)	0.4462 (17)	0.4317 (13)	0.028*	
Cu2	0.41442 (2)	0.11395 (2)	0.46935 (2)	0.01338 (4)	
O3W	0.31197 (13)	0.21798 (8)	0.53582 (6)	0.01739 (17)	
H3A	0.375 (2)	0.2489 (15)	0.5567 (11)	0.021*	
H3B	0.265 (2)	0.2695 (13)	0.5069 (11)	0.021*	
N31	0.18904 (18)	−0.05726 (12)	0.81905 (8)	0.0280 (3)	
H31	0.132 (2)	−0.0769 (17)	0.8677 (10)	0.034*	
C32	0.3521 (2)	−0.06644 (12)	0.81639 (9)	0.0242 (3)	
H32	0.404922	−0.086137	0.870249	0.029*	
C33	0.44317 (17)	−0.04751 (11)	0.73620 (8)	0.0177 (2)	
H33	0.558733	−0.053601	0.734269	0.021*	
C34	0.36311 (16)	−0.01890 (10)	0.65664 (7)	0.0141 (2)	
C35	0.19325 (17)	−0.00897 (12)	0.66241 (9)	0.0200 (2)	
H35	0.136501	0.011228	0.609884	0.024*	
C36	0.1086 (2)	−0.02865 (14)	0.74471 (10)	0.0267 (3)	
H36	−0.006925	−0.022000	0.749028	0.032*	
O34	0.44909 (11)	−0.00094 (7)	0.57869 (5)	0.01311 (15)	
N41	0.0258 (8)	0.3145 (4)	0.2079 (4)	0.0325 (10)	0.466 (10)
H41	−0.056 (4)	0.330 (4)	0.177 (3)	0.039*	0.466 (10)
C42	−0.0027 (6)	0.2731 (4)	0.2963 (4)	0.0266 (9)	0.466 (10)
H42	−0.111579	0.268588	0.322313	0.032*	0.466 (10)
C43	0.1225 (7)	0.2376 (7)	0.3489 (4)	0.0166 (8)	0.466 (10)
H43	0.100103	0.206543	0.410917	0.020*	0.466 (10)
C44	0.2858 (10)	0.2466 (15)	0.3119 (6)	0.0163 (11)	0.466 (10)
O44	0.41538 (13)	0.21725 (8)	0.35668 (6)	0.02085 (19)	0.466 (10)
C45	0.3085 (8)	0.2901 (7)	0.2185 (4)	0.0270 (11)	0.466 (10)
H45	0.415633	0.296640	0.190050	0.032*	0.466 (10)
C46	0.1767 (9)	0.3229 (4)	0.1685 (3)	0.0298 (10)	0.466 (10)
H46	0.193243	0.351543	0.105902	0.036*	0.466 (10)
N41B	0.0829 (7)	0.3253 (4)	0.1787 (3)	0.0366 (9)	0.534 (10)
H41B	0.004 (6)	0.353 (4)	0.143 (3)	0.044*	0.534 (10)
C42B	0.0298 (6)	0.2809 (4)	0.2641 (4)	0.0303 (8)	0.534 (10)
H42B	−0.083800	0.275102	0.280695	0.036*	0.534 (10)
C43B	0.1390 (8)	0.2444 (6)	0.3266 (3)	0.0242 (9)	0.534 (10)
H43B	0.101535	0.213542	0.386922	0.029*	0.534 (10)
C44B	0.3072 (9)	0.2523 (13)	0.3019 (5)	0.0188 (11)	0.534 (10)
O44B	0.41538 (13)	0.21725 (8)	0.35668 (6)	0.02085 (19)	0.534 (10)
C45B	0.3552 (7)	0.3021 (6)	0.2114 (3)	0.0251 (9)	0.534 (10)
H45B	0.467579	0.311888	0.192318	0.030*	0.534 (10)
C46B	0.2419 (8)	0.3354 (4)	0.1529 (3)	0.0336 (9)	0.534 (10)
H46B	0.275068	0.366802	0.092037	0.040*	0.534 (10)
N21	−0.0013 (2)	0.25572 (14)	0.86375 (9)	0.0385 (4)	
H21	−0.022 (3)	0.2076 (17)	0.9120 (11)	0.046*	
C22	0.0892 (2)	0.34613 (16)	0.86027 (10)	0.0340 (4)	
H22	0.130216	0.355965	0.913114	0.041*	
C23	0.1230 (2)	0.42401 (14)	0.78206 (9)	0.0256 (3)	
H23	0.188191	0.487203	0.780589	0.031*	
C24	0.06108 (17)	0.41100 (12)	0.70264 (8)	0.0183 (2)	
C25	−0.0338 (2)	0.31426 (14)	0.71055 (10)	0.0266 (3)	
H25	−0.078311	0.301629	0.659456	0.032*	
C26	−0.0621 (2)	0.23910 (15)	0.79059 (12)	0.0362 (4)	
H26	−0.125545	0.174224	0.794781	0.043*	
O24	0.08872 (14)	0.48390 (9)	0.62773 (6)	0.0235 (2)	
Cl1	0.20336 (4)	−0.12709 (3)	0.44249 (2)	0.01584 (6)	
O1	0.31568 (13)	−0.18345 (8)	0.50809 (7)	0.0234 (2)	
O2	0.18043 (13)	−0.01119 (9)	0.44861 (7)	0.0227 (2)	
O3	0.05148 (13)	−0.18486 (11)	0.46256 (8)	0.0302 (2)	
O4	0.27021 (16)	−0.13051 (11)	0.35540 (7)	0.0325 (3)	
Cl2	0.2533 (5)	−0.3670 (4)	0.9621 (3)	0.0253 (5)	0.428 (3)
O81	0.4083 (9)	−0.3149 (8)	0.9631 (7)	0.0343 (16)	0.428 (3)
O82	0.1212 (4)	−0.2864 (3)	0.95254 (19)	0.0348 (8)	0.428 (3)
O83	0.2681 (5)	−0.4124 (4)	0.8840 (3)	0.0456 (10)	0.428 (3)
O84	0.2197 (4)	−0.4521 (3)	1.0422 (2)	0.0466 (10)	0.428 (3)
Cl2A	0.2771 (7)	−0.3777 (5)	0.9484 (4)	0.0296 (10)	0.429 (3)
O81A	0.2759 (5)	−0.2749 (3)	0.98245 (18)	0.0393 (9)	0.429 (3)
O82A	0.1241 (5)	−0.4324 (4)	0.9797 (2)	0.0495 (10)	0.429 (3)
O83A	0.2862 (16)	−0.3480 (11)	0.8528 (4)	0.0426 (17)	0.429 (3)
O84A	0.4134 (5)	−0.4452 (4)	0.9801 (2)	0.0609 (12)	0.429 (3)
Cl2B	0.278 (2)	−0.3766 (12)	0.9394 (12)	0.030 (2)	0.136 (3)
O81B	0.388 (3)	−0.306 (2)	0.972 (2)	0.028 (3)	0.136 (3)
O82B	0.1181 (15)	−0.3539 (16)	0.9732 (8)	0.052 (3)	0.136 (3)
O83B	0.287 (5)	−0.336 (3)	0.8444 (12)	0.038 (4)	0.136 (3)
O84B	0.3124 (17)	−0.4969 (9)	0.9645 (8)	0.060 (3)	0.136 (3)
Cl3	0.2291 (5)	0.0768 (3)	1.0508 (3)	0.0264 (5)	0.479 (9)
O5	0.2517 (11)	0.0155 (7)	1.1477 (4)	0.0307 (13)	0.479 (9)
O6	0.0765 (10)	0.1351 (6)	1.0496 (5)	0.0371 (11)	0.479 (9)
O7	0.3514 (7)	0.1620 (4)	1.0305 (3)	0.0393 (10)	0.479 (9)
O8	0.2527 (6)	0.0102 (7)	0.9866 (4)	0.0744 (17)	0.479 (9)
Cl3A	0.2365 (4)	0.0482 (4)	1.0563 (2)	0.0276 (6)	0.489 (9)
O5A	0.0761 (10)	0.1029 (7)	1.0399 (6)	0.0491 (17)	0.489 (9)
O6A	0.2447 (13)	0.0073 (8)	1.1428 (5)	0.0497 (19)	0.489 (9)
O7A	0.2326 (7)	−0.0503 (5)	1.0222 (3)	0.0536 (14)	0.489 (9)
O8A	0.3645 (7)	0.1222 (7)	1.0147 (4)	0.0589 (16)	0.489 (9)
Cl3B	0.2444 (19)	0.0223 (16)	1.0312 (11)	0.040 (3)	0.038 (2)
O5B	0.151 (4)	0.028 (3)	1.1156 (16)	0.042 (4)	0.038 (2)
O6B	0.403 (3)	0.063 (3)	1.028 (2)	0.043 (5)	0.038 (2)
O7B	0.248 (6)	−0.094 (2)	1.029 (3)	0.045 (6)	0.038 (2)
O8B	0.174 (4)	0.088 (3)	0.9532 (15)	0.042 (4)	0.038 (2)

### Bis(µ-1,4-dihydropyridin-4-one-κ2O:O)di-µ-perchlorato-κ4O:O'-bis[aqua(1,4-dihydropyridin-4-one-κO)copper(II)] tetraaquabis(1,4-dihydropyridin-4-one-κO)copper(II) tetrakis(perchlorate) 1,4-dihydropyridin-4-one disolvate Atomic displacement parameters (Å^2^)

**Table:**

	*U*^11^	*U*^22^	*U*^33^	*U*^12^	*U*^13^	*U*^23^
Cu1	0.03703 (15)	0.01048 (10)	0.01468 (10)	0.00085 (9)	0.00058 (9)	−0.00184 (7)
N11	0.0415 (9)	0.0435 (9)	0.0158 (5)	−0.0023 (7)	−0.0074 (5)	−0.0072 (5)
C12	0.0363 (9)	0.0408 (10)	0.0270 (7)	−0.0069 (7)	−0.0077 (6)	−0.0157 (7)
C13	0.0276 (8)	0.0238 (7)	0.0233 (6)	−0.0057 (6)	−0.0039 (5)	−0.0077 (5)
C14	0.0214 (6)	0.0161 (6)	0.0169 (5)	0.0014 (5)	−0.0013 (4)	−0.0042 (4)
O14	0.0338 (6)	0.0133 (4)	0.0139 (4)	−0.0010 (4)	−0.0035 (3)	−0.0026 (3)
C15	0.0368 (8)	0.0180 (6)	0.0158 (5)	−0.0009 (6)	−0.0012 (5)	−0.0026 (4)
C16	0.0410 (10)	0.0282 (8)	0.0179 (6)	0.0012 (7)	−0.0014 (6)	−0.0016 (5)
O1W	0.0296 (6)	0.0131 (4)	0.0215 (4)	−0.0009 (4)	0.0011 (4)	−0.0025 (3)
O2W	0.0269 (6)	0.0224 (5)	0.0201 (4)	0.0073 (4)	−0.0024 (4)	−0.0043 (4)
Cu2	0.01613 (8)	0.01182 (7)	0.01180 (6)	0.00301 (5)	−0.00233 (5)	−0.00222 (5)
O3W	0.0191 (5)	0.0149 (4)	0.0195 (4)	0.0035 (4)	−0.0050 (3)	−0.0061 (3)
N31	0.0372 (8)	0.0248 (6)	0.0190 (5)	−0.0034 (6)	0.0121 (5)	−0.0038 (4)
C32	0.0378 (9)	0.0192 (6)	0.0146 (5)	0.0003 (6)	0.0000 (5)	−0.0027 (4)
C33	0.0230 (6)	0.0156 (6)	0.0145 (5)	0.0003 (5)	−0.0026 (4)	−0.0034 (4)
C34	0.0179 (6)	0.0104 (5)	0.0138 (5)	−0.0009 (4)	−0.0004 (4)	−0.0030 (4)
C35	0.0169 (6)	0.0229 (7)	0.0199 (5)	−0.0006 (5)	0.0005 (4)	−0.0050 (5)
C36	0.0226 (7)	0.0273 (8)	0.0287 (7)	−0.0021 (6)	0.0090 (5)	−0.0077 (6)
O34	0.0147 (4)	0.0128 (4)	0.0112 (3)	0.0014 (3)	−0.0005 (3)	−0.0021 (3)
N41	0.034 (2)	0.0360 (18)	0.029 (2)	0.0022 (16)	−0.0186 (16)	−0.0060 (16)
C42	0.028 (2)	0.0252 (17)	0.027 (2)	0.0006 (14)	−0.0056 (15)	−0.0071 (16)
C43	0.0177 (16)	0.0184 (16)	0.0143 (19)	0.0013 (12)	−0.0044 (14)	−0.0041 (18)
C44	0.028 (2)	0.0124 (18)	0.0088 (16)	0.003 (2)	−0.0041 (17)	−0.0017 (16)
O44	0.0260 (5)	0.0183 (5)	0.0156 (4)	0.0051 (4)	−0.0025 (3)	0.0004 (3)
C45	0.030 (3)	0.028 (2)	0.0189 (17)	0.001 (2)	0.0039 (18)	−0.0005 (14)
C46	0.039 (3)	0.0322 (19)	0.0158 (16)	−0.001 (2)	−0.0088 (19)	0.0016 (13)
N41B	0.038 (2)	0.0451 (18)	0.0263 (18)	−0.002 (2)	−0.0176 (18)	−0.0027 (15)
C42B	0.0277 (19)	0.0342 (17)	0.029 (2)	−0.0020 (14)	−0.0109 (15)	−0.0042 (17)
C43B	0.035 (2)	0.0202 (15)	0.0159 (19)	−0.0004 (14)	−0.0029 (16)	−0.0018 (18)
C44B	0.026 (2)	0.015 (2)	0.016 (2)	0.0045 (18)	−0.0015 (14)	−0.0054 (18)
O44B	0.0260 (5)	0.0183 (5)	0.0156 (4)	0.0051 (4)	−0.0025 (3)	0.0004 (3)
C45B	0.035 (2)	0.0263 (19)	0.0105 (12)	0.0025 (19)	−0.0003 (15)	0.0023 (11)
C46B	0.042 (2)	0.040 (2)	0.0178 (15)	0.0021 (19)	−0.0062 (15)	−0.0042 (13)
N21	0.0498 (10)	0.0332 (8)	0.0227 (6)	0.0058 (7)	0.0106 (6)	0.0061 (5)
C22	0.0411 (10)	0.0419 (10)	0.0186 (6)	0.0085 (8)	−0.0052 (6)	−0.0067 (6)
C23	0.0278 (8)	0.0282 (8)	0.0232 (6)	0.0005 (6)	−0.0047 (5)	−0.0102 (5)
C24	0.0186 (6)	0.0181 (6)	0.0179 (5)	0.0014 (5)	−0.0006 (4)	−0.0044 (4)
C25	0.0299 (8)	0.0246 (7)	0.0269 (7)	−0.0066 (6)	−0.0003 (5)	−0.0088 (5)
C26	0.0416 (10)	0.0245 (8)	0.0393 (9)	−0.0081 (7)	0.0117 (7)	−0.0056 (7)
O24	0.0283 (6)	0.0204 (5)	0.0190 (4)	0.0004 (4)	−0.0003 (4)	−0.0003 (3)
Cl1	0.01294 (13)	0.01720 (13)	0.01787 (12)	−0.00132 (10)	−0.00246 (9)	−0.00465 (10)
O1	0.0232 (5)	0.0136 (4)	0.0332 (5)	0.0002 (4)	−0.0138 (4)	−0.0017 (4)
O2	0.0204 (5)	0.0177 (5)	0.0308 (5)	0.0056 (4)	−0.0068 (4)	−0.0067 (4)
O3	0.0157 (5)	0.0356 (7)	0.0393 (6)	−0.0099 (5)	−0.0012 (4)	−0.0082 (5)
O4	0.0420 (7)	0.0373 (7)	0.0207 (5)	−0.0118 (6)	0.0067 (4)	−0.0134 (4)
Cl2	0.0302 (14)	0.0283 (8)	0.0172 (9)	−0.0050 (8)	−0.0085 (7)	−0.0023 (6)
O81	0.034 (3)	0.054 (3)	0.017 (2)	−0.016 (2)	−0.0017 (19)	−0.0120 (17)
O82	0.0373 (17)	0.0387 (19)	0.0251 (13)	0.0062 (14)	−0.0060 (11)	−0.0011 (12)
O83	0.049 (2)	0.059 (3)	0.043 (2)	−0.0015 (19)	−0.0142 (16)	−0.0370 (18)
O84	0.0432 (19)	0.0417 (19)	0.0428 (17)	−0.0134 (15)	−0.0115 (14)	0.0171 (13)
Cl2A	0.0257 (15)	0.0374 (16)	0.026 (2)	−0.0016 (11)	−0.0047 (12)	−0.0068 (12)
O81A	0.052 (2)	0.0420 (18)	0.0266 (13)	−0.0069 (16)	−0.0090 (13)	−0.0114 (12)
O82A	0.047 (2)	0.056 (2)	0.0437 (18)	−0.0236 (19)	0.0031 (15)	−0.0083 (17)
O83A	0.041 (3)	0.057 (4)	0.027 (2)	−0.010 (3)	−0.006 (2)	−0.002 (3)
O84A	0.050 (2)	0.074 (3)	0.053 (2)	0.031 (2)	−0.0158 (17)	−0.0048 (19)
Cl2B	0.025 (3)	0.032 (3)	0.026 (3)	−0.001 (3)	−0.003 (2)	0.004 (2)
O81B	0.035 (6)	0.035 (5)	0.018 (6)	−0.004 (5)	0.001 (5)	−0.011 (4)
O82B	0.043 (5)	0.069 (6)	0.040 (5)	0.012 (6)	0.004 (4)	−0.009 (5)
O83B	0.041 (7)	0.048 (7)	0.021 (6)	−0.004 (6)	−0.001 (5)	0.000 (6)
O84B	0.065 (6)	0.035 (5)	0.070 (6)	0.001 (5)	−0.012 (5)	0.011 (4)
Cl3	0.0321 (7)	0.0309 (10)	0.0171 (8)	−0.0071 (8)	0.0033 (5)	−0.0086 (8)
O5	0.033 (3)	0.047 (3)	0.0125 (16)	−0.012 (2)	−0.0036 (16)	−0.0060 (18)
O6	0.0345 (19)	0.052 (3)	0.0202 (16)	−0.006 (2)	−0.0008 (12)	0.0000 (17)
O7	0.045 (2)	0.038 (2)	0.0329 (17)	−0.0166 (17)	0.0086 (13)	−0.0062 (13)
O8	0.098 (3)	0.085 (4)	0.059 (3)	−0.037 (3)	0.029 (2)	−0.058 (3)
Cl3A	0.0221 (6)	0.0425 (15)	0.0156 (5)	−0.0072 (9)	−0.0001 (4)	−0.0016 (9)
O5A	0.037 (2)	0.072 (4)	0.027 (2)	0.005 (3)	−0.0033 (15)	0.007 (2)
O6A	0.041 (4)	0.039 (3)	0.052 (3)	0.001 (3)	0.011 (3)	0.019 (3)
O7A	0.072 (3)	0.063 (3)	0.033 (2)	−0.009 (2)	−0.0006 (16)	−0.026 (2)
O8A	0.047 (2)	0.079 (4)	0.040 (2)	−0.035 (3)	0.0146 (17)	0.004 (2)
Cl3B	0.042 (4)	0.051 (4)	0.024 (4)	−0.010 (4)	0.002 (4)	−0.003 (4)
O5B	0.042 (7)	0.055 (7)	0.023 (6)	−0.005 (7)	0.005 (6)	0.000 (6)
O6B	0.041 (8)	0.050 (8)	0.034 (8)	−0.026 (8)	0.018 (7)	−0.007 (7)
O7B	0.050 (10)	0.054 (10)	0.032 (9)	−0.019 (9)	0.009 (9)	−0.014 (9)
O8B	0.046 (7)	0.055 (7)	0.025 (7)	−0.017 (7)	0.005 (7)	−0.006 (7)

### Bis(µ-1,4-dihydropyridin-4-one-κ2O:O)di-µ-perchlorato-κ4O:O'-bis[aqua(1,4-dihydropyridin-4-one-κO)copper(II)] tetraaquabis(1,4-dihydropyridin-4-one-κO)copper(II) tetrakis(perchlorate) 1,4-dihydropyridin-4-one disolvate Geometric parameters (Å, º)

**Table:**

Cu1—O14^i^	1.9276 (9)	C46—H46	0.9500
Cu1—O14	1.9276 (9)	N41B—C46B	1.339 (5)
Cu1—O1W	1.9501 (11)	N41B—C42B	1.346 (5)
Cu1—O1W^i^	1.9501 (11)	N41B—H41B	0.89 (5)
Cu1—O2W	2.8760 (12)	C42B—C43B	1.363 (6)
N11—C12	1.341 (2)	C42B—H42B	0.9500
N11—C16	1.351 (2)	C43B—C44B	1.406 (7)
N11—H11	0.822 (15)	C43B—H43B	0.9500
C12—C13	1.363 (2)	C44B—O44B	1.273 (7)
C12—H12	0.9500	C44B—C45B	1.418 (7)
C13—C14	1.4193 (18)	C45B—C46B	1.340 (6)
C13—H13	0.9500	C45B—H45B	0.9500
C14—O14	1.2965 (15)	C46B—H46B	0.9500
C14—C15	1.4130 (19)	N21—C22	1.341 (3)
C15—C16	1.3588 (19)	N21—C26	1.343 (2)
C15—H15	0.9500	N21—H21	0.839 (15)
C16—H16	0.9500	C22—C23	1.361 (2)
O1W—H1A	0.779 (15)	C22—H22	0.9500
O1W—H1B	0.794 (14)	C23—C24	1.4211 (18)
O2W—H2A	0.788 (14)	C23—H23	0.9500
O2W—H2B	0.83 (2)	C24—O24	1.2885 (16)
Cu2—O44B	1.8895 (9)	C24—C25	1.414 (2)
Cu2—O44	1.8895 (9)	C25—C26	1.360 (2)
Cu2—O3W	1.9534 (10)	C25—H25	0.9500
Cu2—O34	1.9534 (9)	C26—H26	0.9500
Cu2—O34^ii^	1.9943 (9)	Cl1—O4	1.4290 (10)
Cu2—O1^ii^	2.4885 (10)	Cl1—O3	1.4312 (11)
Cu2—O2	2.5898 (10)	Cl1—O2	1.4453 (10)
Cu2—Cu2^ii^	3.0427 (3)	Cl1—O1	1.4609 (10)
O3W—H3A	0.786 (13)	Cl2—O84	1.426 (5)
O3W—H3B	0.787 (14)	Cl2—O82	1.431 (5)
N31—C32	1.341 (2)	Cl2—O83	1.448 (5)
N31—C36	1.345 (2)	Cl2—O81	1.449 (7)
N31—H31	0.843 (14)	Cl2A—O84A	1.416 (7)
C32—C33	1.3733 (18)	Cl2A—O82A	1.438 (7)
C32—H32	0.9500	Cl2A—O83A	1.438 (6)
C33—C34	1.4121 (17)	Cl2A—O81A	1.477 (6)
C33—H33	0.9500	Cl2B—O82B	1.418 (13)
C34—O34	1.3254 (14)	Cl2B—O83B	1.435 (13)
C34—C35	1.3964 (19)	Cl2B—O84B	1.447 (13)
C35—C36	1.3762 (19)	Cl2B—O81B	1.472 (14)
C35—H35	0.9500	Cl3—O6	1.420 (8)
C36—H36	0.9500	Cl3—O8	1.433 (5)
N41—C46	1.335 (6)	Cl3—O7	1.434 (5)
N41—C42	1.347 (5)	Cl3—O5	1.532 (7)
N41—H41	0.844 (19)	Cl3A—O6A	1.322 (7)
C42—C43	1.361 (6)	Cl3A—O8A	1.417 (5)
C42—H42	0.9500	Cl3A—O7A	1.430 (5)
C43—C44	1.414 (8)	Cl3A—O5A	1.471 (8)
C43—H43	0.9500	Cl3B—O6B	1.410 (14)
C44—O44	1.313 (7)	Cl3B—O7B	1.425 (14)
C44—C45	1.419 (8)	Cl3B—O8B	1.433 (14)
C45—C46	1.377 (7)	Cl3B—O5B	1.482 (14)
C45—H45	0.9500		
			
O14^i^—Cu1—O14	180.0	C44—C43—H43	119.8
O14^i^—Cu1—O1W	88.92 (4)	O44—C44—C43	125.4 (6)
O14—Cu1—O1W	91.08 (4)	O44—C44—C45	118.3 (6)
O14^i^—Cu1—O1W^i^	91.08 (4)	C43—C44—C45	116.2 (5)
O14—Cu1—O1W^i^	88.92 (4)	C44—O44—Cu2	124.3 (4)
O1W—Cu1—O1W^i^	180.0	C46—C45—C44	120.7 (5)
O14^i^—Cu1—O2W	99.35 (4)	C46—C45—H45	119.7
O14—Cu1—O2W	80.65 (4)	C44—C45—H45	119.7
O1W—Cu1—O2W	81.98 (4)	N41—C46—C45	120.1 (4)
O1W^i^—Cu1—O2W	98.02 (4)	N41—C46—H46	120.0
C12—N11—C16	121.44 (13)	C45—C46—H46	120.0
C12—N11—H11	117.4 (15)	C46B—N41B—C42B	121.7 (4)
C16—N11—H11	121.2 (15)	C46B—N41B—H41B	124 (3)
N11—C12—C13	121.39 (13)	C42B—N41B—H41B	114 (3)
N11—C12—H12	119.3	N41B—C42B—C43B	120.1 (4)
C13—C12—H12	119.3	N41B—C42B—H42B	120.0
C12—C13—C14	119.42 (14)	C43B—C42B—H42B	120.0
C12—C13—H13	120.3	C42B—C43B—C44B	120.0 (4)
C14—C13—H13	120.3	C42B—C43B—H43B	120.0
O14—C14—C15	119.06 (11)	C44B—C43B—H43B	120.0
O14—C14—C13	124.11 (12)	O44B—C44B—C43B	123.0 (6)
C15—C14—C13	116.82 (12)	O44B—C44B—C45B	119.7 (6)
C14—O14—Cu1	130.66 (8)	C43B—C44B—C45B	117.3 (5)
C16—C15—C14	121.04 (13)	C44B—O44B—Cu2	133.8 (4)
C16—C15—H15	119.5	C46B—C45B—C44B	119.9 (5)
C14—C15—H15	119.5	C46B—C45B—H45B	120.0
N11—C16—C15	119.88 (15)	C44B—C45B—H45B	120.0
N11—C16—H16	120.1	N41B—C46B—C45B	121.1 (4)
C15—C16—H16	120.1	N41B—C46B—H46B	119.5
Cu1—O1W—H1A	118.0 (14)	C45B—C46B—H46B	119.5
Cu1—O1W—H1B	116.5 (14)	C22—N21—C26	121.31 (14)
H1A—O1W—H1B	109 (2)	C22—N21—H21	120.8 (15)
Cu1—O2W—H2A	89.2 (14)	C26—N21—H21	117.9 (15)
Cu1—O2W—H2B	136.3 (13)	N21—C22—C23	120.94 (14)
H2A—O2W—H2B	108 (2)	N21—C22—H22	119.5
O44B—Cu2—O3W	95.60 (4)	C23—C22—H22	119.5
O44—Cu2—O3W	95.60 (4)	C22—C23—C24	120.16 (14)
O44B—Cu2—O34	170.44 (4)	C22—C23—H23	119.9
O44—Cu2—O34	170.44 (4)	C24—C23—H23	119.9
O3W—Cu2—O34	91.88 (4)	O24—C24—C25	121.88 (12)
O44B—Cu2—O34^ii^	92.58 (4)	O24—C24—C23	121.82 (13)
O44—Cu2—O34^ii^	92.58 (4)	C25—C24—C23	116.30 (13)
O3W—Cu2—O34^ii^	168.13 (4)	C26—C25—C24	120.65 (14)
O34—Cu2—O34^ii^	79.16 (4)	C26—C25—H25	119.7
O44—Cu2—O1^ii^	90.09 (4)	C24—C25—H25	119.7
O3W—Cu2—O1^ii^	88.40 (4)	N21—C26—C25	120.64 (16)
O34—Cu2—O1^ii^	84.16 (4)	N21—C26—H26	119.7
O34^ii^—Cu2—O1^ii^	82.98 (4)	C25—C26—H26	119.7
O44B—Cu2—O2	97.32 (4)	O4—Cl1—O3	110.20 (7)
O44—Cu2—O2	97.32 (4)	O4—Cl1—O2	110.13 (7)
O3W—Cu2—O2	105.16 (4)	O3—Cl1—O2	110.18 (7)
O34—Cu2—O2	86.43 (3)	O4—Cl1—O1	109.55 (8)
O34^ii^—Cu2—O2	82.24 (3)	O3—Cl1—O1	108.33 (7)
O1^ii^—Cu2—O2	163.73 (3)	O2—Cl1—O1	108.40 (6)
O44—Cu2—Cu2^ii^	131.50 (3)	Cl1—O2—Cu2	123.65 (6)
O3W—Cu2—Cu2^ii^	131.49 (3)	O84—Cl2—O82	108.4 (3)
O34—Cu2—Cu2^ii^	40.07 (3)	O84—Cl2—O83	112.5 (4)
O34^ii^—Cu2—Cu2^ii^	39.09 (2)	O82—Cl2—O83	107.9 (4)
O1^ii^—Cu2—Cu2^ii^	81.64 (2)	O84—Cl2—O81	110.6 (5)
O2—Cu2—Cu2^ii^	82.61 (2)	O82—Cl2—O81	112.1 (5)
Cu2—O3W—H3A	113.4 (13)	O83—Cl2—O81	105.5 (4)
Cu2—O3W—H3B	114.6 (13)	O84A—Cl2A—O82A	113.1 (5)
H3A—O3W—H3B	101.6 (18)	O84A—Cl2A—O83A	110.8 (6)
C32—N31—C36	122.10 (12)	O82A—Cl2A—O83A	107.7 (6)
C32—N31—H31	120.8 (14)	O84A—Cl2A—O81A	107.8 (5)
C36—N31—H31	116.7 (14)	O82A—Cl2A—O81A	106.9 (4)
N31—C32—C33	120.33 (13)	O83A—Cl2A—O81A	110.4 (6)
N31—C32—H32	119.8	O82B—Cl2B—O83B	108.1 (18)
C33—C32—H32	119.8	O82B—Cl2B—O84B	109.9 (13)
C32—C33—C34	119.26 (13)	O83B—Cl2B—O84B	110.6 (17)
C32—C33—H33	120.4	O82B—Cl2B—O81B	106.4 (15)
C34—C33—H33	120.4	O83B—Cl2B—O81B	106.3 (18)
O34—C34—C35	121.41 (11)	O84B—Cl2B—O81B	115.3 (16)
O34—C34—C33	120.04 (12)	O6—Cl3—O8	114.0 (4)
C35—C34—C33	118.54 (11)	O6—Cl3—O7	106.2 (4)
C36—C35—C34	119.53 (13)	O8—Cl3—O7	108.1 (3)
C36—C35—H35	120.2	O6—Cl3—O5	107.3 (5)
C34—C35—H35	120.2	O8—Cl3—O5	116.6 (4)
N31—C36—C35	120.22 (15)	O7—Cl3—O5	103.7 (5)
N31—C36—H36	119.9	O6A—Cl3A—O8A	114.4 (5)
C35—C36—H36	119.9	O6A—Cl3A—O7A	104.0 (4)
C34—O34—Cu2	128.72 (8)	O8A—Cl3A—O7A	113.1 (4)
C34—O34—Cu2^ii^	126.89 (8)	O6A—Cl3A—O5A	109.1 (6)
Cu2—O34—Cu2^ii^	100.84 (4)	O8A—Cl3A—O5A	111.3 (5)
C46—N41—C42	121.7 (4)	O7A—Cl3A—O5A	104.3 (4)
C46—N41—H41	121 (3)	O6B—Cl3B—O7B	111 (2)
C42—N41—H41	117 (3)	O6B—Cl3B—O8B	106.2 (17)
N41—C42—C43	120.9 (4)	O7B—Cl3B—O8B	109.1 (19)
N41—C42—H42	119.5	O6B—Cl3B—O5B	110.5 (17)
C43—C42—H42	119.5	O7B—Cl3B—O5B	105.8 (18)
C42—C43—C44	120.4 (5)	O8B—Cl3B—O5B	114.0 (18)
C42—C43—H43	119.8		
			
C16—N11—C12—C13	−0.1 (3)	Cu2^ii^—Cu2—O44—C44	117.7 (10)
N11—C12—C13—C14	1.0 (3)	O44—C44—C45—C46	−179.5 (11)
C12—C13—C14—O14	179.73 (16)	C43—C44—C45—C46	1.1 (18)
C12—C13—C14—C15	−1.0 (2)	C42—N41—C46—C45	−0.8 (9)
C15—C14—O14—Cu1	174.04 (11)	C44—C45—C46—N41	0.3 (12)
C13—C14—O14—Cu1	−6.7 (2)	C46B—N41B—C42B—C43B	−0.4 (8)
O14—C14—C15—C16	179.57 (15)	N41B—C42B—C43B—C44B	−0.4 (11)
C13—C14—C15—C16	0.3 (2)	C42B—C43B—C44B—O44B	−178.3 (11)
C12—N11—C16—C15	−0.7 (3)	C42B—C43B—C44B—C45B	1.8 (16)
C14—C15—C16—N11	0.6 (3)	C43B—C44B—O44B—Cu2	20 (2)
C36—N31—C32—C33	−0.6 (2)	C45B—C44B—O44B—Cu2	−159.9 (6)
N31—C32—C33—C34	−0.3 (2)	O3W—Cu2—O44B—C44B	−78.7 (10)
C32—C33—C34—O34	−179.65 (12)	O34^ii^—Cu2—O44B—C44B	109.9 (10)
C32—C33—C34—C35	1.06 (18)	O1^ii^—Cu2—O44B—C44B	−167.1 (10)
O34—C34—C35—C36	179.83 (12)	O2—Cu2—O44B—C44B	27.4 (10)
C33—C34—C35—C36	−0.89 (19)	Cu2^ii^—Cu2—O44B—C44B	114.0 (10)
C32—N31—C36—C35	0.8 (2)	O44B—C44B—C45B—C46B	177.6 (10)
C34—C35—C36—N31	0.0 (2)	C43B—C44B—C45B—C46B	−2.5 (16)
C35—C34—O34—Cu2	39.13 (16)	C42B—N41B—C46B—C45B	−0.3 (8)
C33—C34—O34—Cu2	−140.14 (10)	C44B—C45B—C46B—N41B	1.8 (11)
C35—C34—O34—Cu2^ii^	−115.41 (12)	C26—N21—C22—C23	−0.3 (3)
C33—C34—O34—Cu2^ii^	65.32 (13)	N21—C22—C23—C24	0.6 (3)
C46—N41—C42—C43	−0.3 (8)	C22—C23—C24—O24	178.98 (15)
N41—C42—C43—C44	1.8 (12)	C22—C23—C24—C25	−0.4 (2)
C42—C43—C44—O44	178.5 (12)	O24—C24—C25—C26	−179.49 (16)
C42—C43—C44—C45	−2.2 (17)	C23—C24—C25—C26	−0.1 (2)
C43—C44—O44—Cu2	22 (2)	C22—N21—C26—C25	−0.2 (3)
C45—C44—O44—Cu2	−157.6 (8)	C24—C25—C26—N21	0.4 (3)
O3W—Cu2—O44—C44	−75.0 (10)	O4—Cl1—O2—Cu2	−78.50 (8)
O34^ii^—Cu2—O44—C44	113.6 (10)	O3—Cl1—O2—Cu2	159.73 (6)
O1^ii^—Cu2—O44—C44	−163.4 (10)	O1—Cl1—O2—Cu2	41.34 (8)
O2—Cu2—O44—C44	31.1 (10)		

Symmetry codes: (i) −*x*+1, −*y*+1, −*z*+1; (ii) −*x*+1, −*y*, −*z*+1.

### Bis(µ-1,4-dihydropyridin-4-one-κ2O:O)di-µ-perchlorato-κ4O:O'-bis[aqua(1,4-dihydropyridin-4-one-κO)copper(II)] tetraaquabis(1,4-dihydropyridin-4-one-κO)copper(II) tetrakis(perchlorate) 1,4-dihydropyridin-4-one disolvate Hydrogen-bond geometry (Å, º)

**Table:**

*D*—H···*A*	*D*—H	H···*A*	*D*···*A*	*D*—H···*A*
N11—H11···O81^iii^	0.82 (2)	2.06 (2)	2.858 (9)	164 (2)
N11—H11···O81*A*^iii^	0.82 (2)	2.26 (2)	2.961 (3)	143 (2)
O1*W*—H1*A*···O1^iv^	0.78 (2)	1.99 (2)	2.7695 (15)	176 (2)
O1*W*—H1*B*···O24	0.79 (1)	1.83 (2)	2.6124 (15)	171 (2)
O2*W*—H2*A*···O24	0.79 (1)	1.99 (1)	2.7753 (14)	172 (2)
O2*W*—H2*B*···O24^v^	0.83 (2)	2.07 (2)	2.8705 (16)	161 (2)
O3*W*—H3*A*···O14	0.79 (1)	1.94 (1)	2.6941 (13)	161 (2)
O3*W*—H3*B*···O2*W*	0.79 (1)	1.95 (2)	2.6875 (15)	157 (2)
N31—H31···O6^vi^	0.84 (1)	2.09 (2)	2.882 (9)	156 (2)
N31—H31···O5*A*^vi^	0.84 (1)	2.12 (2)	2.928 (9)	160 (2)
N41—H41···O83^vii^	0.84 (2)	2.16 (2)	2.974 (6)	163 (5)
N41*B*—H41*B*···O82*A*^vii^	0.89 (5)	2.25 (5)	3.104 (6)	161 (4)
N41*B*—H41*B*···O83*A*^vii^	0.89 (5)	2.39 (5)	3.109 (14)	138 (4)
N21—H21···O6	0.84 (2)	2.31 (2)	3.013 (9)	142 (2)
N21—H21···O5*A*	0.84 (2)	2.28 (2)	3.013 (9)	147 (2)
N21—H21···O7*A*^vi^	0.84 (2)	2.59 (2)	3.263 (7)	138 (2)

Symmetry codes: (iii) −*x*+1, −*y*, −*z*+2; (iv) *x*, *y*+1, *z*; (v) −*x*, −*y*+1, −*z*+1; (vi) −*x*, −*y*, −*z*+2; (vii) −*x*, −*y*, −*z*+1.
